# Multimorbidity in the Brazilian adult population: Protocol for a systematic review and meta-analysis of prevalence

**DOI:** 10.1371/journal.pone.0343166

**Published:** 2026-03-04

**Authors:** Cristina Camargo Pereira, Larissa Silva Magalhães, Sandro Rogério Rodrigues Batista, Valéria Pagotto, Rafael Alves Guimarães

**Affiliations:** 1 Institute of Tropical Pathology and Public Health, Federal University of Goiás, Goiânia, Goiás, Brazil; 2 College of Nursing, Auburn University, Auburn, Alabama, United States of America; 3 Faculty of Nursing, Federal University of Goiás, Goiânia, Goiás, Brazil; 4 Faculty of Medicine, Federal University of Goiás, Goiânia, Goiás, Brazil; 5 Postgraduate Program in Medical Sciences, Faculty of Medicine, University of Brasília, Brasília, Brazil; McMaster University, CANADA

## Abstract

Multimorbidity (MM), defined as the co-occurrence of multiple chronic conditions in a single individual, poses a major challenge to health systems. Its consequences include higher morbidity and mortality rates, reduced quality of life, and increased healthcare costs. Despite its substantial public health burden, no systematic reviews have comprehensively assessed the pooled prevalence of MM in Brazil. This manuscript outlines a protocol for a systematic review and meta-analysis aimed at estimating the prevalence of MM among community-dwelling adults in Brazil. We will conduct a systematic review and meta-analysis of population-based studies reporting MM prevalence in community settings. A comprehensive search will be performed in PubMed, Scopus, Web of Science, Embase, LILACS, and SciELO databases. Two independent reviewers will screen articles, assess study quality using the Joanna Briggs Institute (JBI) Checklist for prevalence studies, and extract data. For the meta-analysis, pooled estimates will be calculated using random-effects models with Restricted Maximum Likelihood (REML) estimators to account for between-study variability. Heterogeneity will be assessed using the I² statistic and Cochran’s Q test. Subgroups analyses (e.g., age group, sex, region, and study type) will be conducted where feasible. Findings will be reported following the Preferred Reporting Items for Systematic Reviews and Meta-Analyses (PRISMA) guidelines. The protocol is registered with the International Prospective Register of Systematic Reviews (CRD42024389106). This review will provide comprehensive evidence on MM prevalence in Brazil, identifying the burden of this problem for future research and informing public health strategies.

## Introduction

Population aging, driven by increased life expectancy and declining fertility rates, has led to an increased prevalence of chronic conditions globally [1,2]. Advances in public health have extended lifespan, resulting in a growing proportion of individuals living with multiple, complex chronic conditions [3]. Concurrently, unhealthy lifestyles – such as physical inactivity [4] and poor dietary habits [5] – coupled with adverse socioeconomic (e.g., low income and education) [6] and environmental determinants (e.g., air pollution, extreme temperatures) [7], exacerbating the burden of diseases such as diabetes, cancer, and cardiovascular diseases (CVD) [8].

Multimorbidity (MM), defined as the coexistence of multiple chronic conditions in a single individual [9], is associated with significant adverse outcomes [10]. These include higher hospitalization rates [11], increased dependence on long-term care [12], functional disability (particularly among women) [13,14], diminished quality of life [15], elevated healthcare costs [16], and premature mortality [17]. This phenomenon poses a mounting challenge to healthcare systems worldwide [18], exacerbated by ongoing demographic, nutritional, and epidemiological transitions [19].

While MM is a global health concern, its prevalence varies substantially between populations, as demonstrated by previous systematic reviews [20–25]. This heterogeneity stems from methodological differences, including variations in: (i) target population (e.g., sex and age group), (ii) definitional approaches of MM (e.g., simple counts versus weighted indices for classification), and (iii) assessment methods (e.g., clinically measured versus self-reported conditions) [25–27].

The most common widely accepted definition of MM requires the presence of two or more chronic conditions in an individual [9], though operational definitions vary from two to five or more conditions [28]. Notably, there remains no consensus regarding which specific chronic conditions should be included in the MM definition [29]. Measurement approaches range from simple condition counts to weighted indices that account for disease severity and individual impact [30].

A recent meta-analysis reported substantial geographic variation in MM prevalence among community-dwelling adults: 45.7% in South America, 43.1% in North America, 39.2% in Europe, and 35.0% in Asia, with an overall global prevalence of 37.2% [20]. Another meta-analysis focusing on Latin American and Caribbean countries (excluding Brazil) found a prevalence of 35.0% [22]. In Brazil, where 20 studies were analyzed, the pooled prevalence reached 50.0% [22].

Population-based studies in Brazil have reported MM prevalence ranging from 10.9 to 42.5% among adults and elderly populations [31–33]. Previous systematic reviews that included data from Brazil have several limitations. For example, they did not incorporate recent studies conducted on adult community populations. Furthermore, they did not perform a detailed analysis of the updated prevalence of MM in Brazilian adults and the elderly, disaggregated by population subgroups (age group and sex) and geographic regions (Brazilian regions), which hinders the development of strategies and public policies to reduce this phenomenon [22]. Lastly, there is a gap regarding the trend of MM over the years in the country in systematic reviews with Brazilian data. Thus, we aim to conduct a systematic review and meta-analysis to estimate the prevalence of MM in the Brazilian adult population.

## Materials and methods

### Protocol and registration

This systematic review protocol was registered in the International Prospective Register of Systematic Reviews (PROSPERO) under registration number CRD42024389106. Any modifications to the protocol will be documented in PROSPERO and reported in the final manuscript. This report follows the Preferred Reporting Items for Systematic Reviews and Meta-Analyses (PRISMA) guidelines (Supplementary Material in S1 File) [34].

To structure the review, we applied the CoCoPop mnemonic [35]. The condition of interest (Co) is MM, while the context (Co) is Brazil, including national and subnational studies. The population (Pop) comprises adults aged 18 years or older of both sexes, derived from representative population-based studies of community-dwelling Brazilian adults. The research question guiding this review is: *What is the prevalence of MM in the Brazilian adult community-dwelling population?*

### Status and timeline of the study

The study is currently in the article selection phase. We anticipate completing this phase by August 2025, followed by data extraction, which should be finalized by September 2025. Results are expected to be available by December 2026.

### Definitions

We adopt the World Health Organization’s definition of MM as the coexistence of two or more chronic conditions in the same individual [9]. However, definitions vary substantially across studies depending on the number, type, and assessment method of conditions. A previous systematic review found that the number of conditions considered ranged from 2 to 285 (median 17, Interquartile range [IQR]: 11–23), with most studies focusing on eight key conditions: diabetes, stroke, cancer, Chronic Obstructive Pulmonary Disease (COPD), hypertension, coronary heart disease, chronic kidney disease, and heart failure [21].

The literature also highlights discrepancies between simple counts of conditions and weighted indices that account for condition severity or impact [28]. Nevertheless, systematic reviews suggest that both approaches are equally effective in predicting adverse outcomes associated with MM [28,30,36]. Therefore, this review will consider both methods.

### Eligibility criteria

Included studies must meet the following criteria: (1) define MM as two or more (≥2) chronic conditions [37]; (2) report MM as either an outcome or an exposure variable; (3) assess conditions through self-report, clinical measurement, or both; (4) provide a list of at least five chronic conditions used to define MM; (5) be cross-sectional or cohort studies with probabilistic sampling; (6) include adults aged 18 years or older of both sexes; (7) cover municipal, regional, or national populations; and (8) be conducted in community settings. If multiple studies analyze the same population, only the earliest publication will be included. For cohort studies, only baseline prevalence data will be extracted.

Studies will be excluded if they: (1) focus on specific subgroups such as Indigenous populations, pregnant women, or patients with pre-specified conditions like hypertension; (2) do not specify the cutoff for defining MM; (3) fail to provide the list of chronic conditions; (4) include only acute conditions; (5) study hospitalized or outpatient populations in primary or hospital care settings; (6) analyze institutionalized populations such as nursing home residents; (7) use sub-samples from larger surveys; or (8) are qualitative studies, case-control studies, interventional studies, opinion pieces, conference abstracts, books, letters, editorials, reviews, or theses/dissertations.

### Search strategy

We will search the following databases: MEDLINE (MEDical Literature Analysis and Retrieval System) accessed via PubMed (Public Medical Database), Scopus, Web of Science accessed via Portal CAPES (Coordination for the Improvement of Higher Education Personnel), Embase (Excerpta Medica Database), LILACS (Latin American and Caribbean Health Sciences Literature) accessed via the Virtual Health Library (VHL), and SciELO (Scientific Electronic Library Online).

The search strategy includes Medical Subject Headings (MeSH) terms such as “prevalence”, “epidemiology”, “multimorbidity”, “multiple chronic conditions,” and “Brazil”, along with uncontrolled terms like “surveillance” and “Brazilian” to enhance sensitivity (Table 1). The selection of descriptors and terms was based on analyzing the research question, previous systematic reviews, and the consultation of synonyms.

**Table 1:** List of the main terms used in the search strategy.

1	Prevalence OR Epidemiology OR Surveillance
2	Multimorbidity OR “multi morbid*” OR “multimorbid* OR “multiple chronic conditions”
3	Brazil OR Brazilian
4	1 AND 2 AND 3

The search strategy was initially developed in PubMed and reviewed by a subject-matter expert. Controlled descriptors were combined with free-text terms. The strategy was designed using a combination of Boolean operators AND and OR and truncated terms for “multimorbidity” (multi morbid*, multimorbid*) (Table 1). Subsequently, the strategy was adapted for each specific database. No language or publication date restrictions were applied. The search strategies are detailed in Tables 2–7.

**Table 2:** PubMed search strategy.

Search	Query
#1	Prevalence[MeSH Terms]
#2	Prevalence[Title/Abstract]
#3	Epidemiology[MeSH Terms]
#4	Epidemiology[Title/Abstract]
#5	Surveillance[Title/Abstract]
#6	“Prevalence”[MeSH Terms] OR “Prevalence”[Title/Abstract] OR “Epidemiology”[MeSH Terms] OR “Epidemiology”[Title/Abstract] OR “Surveillance”[Title/Abstract]
#7	“multimorbidity”[MeSH Terms]
#8	“multi morbid*”[Title/Abstract]
#9	“multimorbid*”[Title/Abstract]
#10	“multiple chronic conditions”[MeSH Terms]
#11	“multiple chronic conditions”[Title/Abstract]
#12	“multimorbidity”[MeSH Terms] OR “multi morbid*”[Title/Abstract] OR “multimorbid*”[Title/Abstract] OR “multiple chronic conditions”[MeSH Terms] OR “multiple chronic conditions”[Title/Abstract]
#13	“Brazil”[MeSH Terms]
#14	“Brazil”[Title/Abstract]
#15	“brazilian”[Title/Abstract]
#16	“Brazil”[MeSH Terms] OR “Brazil”[Title/Abstract] OR “brazilian”[Title/Abstract]
#17	(“Prevalence”[MeSH Terms] OR “Prevalence”[Title/Abstract] OR “Epidemiology”[MeSH Terms] OR “Epidemiology”[Title/Abstract] OR “Surveillance”[Title/Abstract]) AND (“multimorbidity”[MeSH Terms] OR “multi morbid*”[Title/Abstract] OR “multimorbid*”[Title/Abstract] OR “multiple chronic conditions”[MeSH Terms] OR “multiple chronic conditions”[Title/Abstract]) AND (“Brazil”[MeSH Terms] OR “Brazil”[Title/Abstract] OR “brazilian”[Title/Abstract] OR (“Brazil”[MeSH Terms] OR “Brazil”[Title/Abstract] OR “brazilian”[Title/Abstract]))

**Table 3:** Scopus search strategy.

Search	Query
#1	INDEXTERMS (prevalence)
#2	TITLE-ABS-KEY (prevalence)
#3	INDEXTERMS (epidemiology)
#4	TITLE-ABS-KEY(epidemiology)
#5	TITLE-ABS-KEY (surveillance)
#6	INDEXTERMS (prevalence) OR TITLE-ABS-KEY (prevalence) OR INDEXTERMS (epidemiology) OR TITLE-ABS-KEY (epidemiology) OR TITLE-ABS-KEY (surveillance)
#7	INDEXTERMS (multimorbidity)
#8	TITLE-ABS-KEY (“multi morbid*”)
#9	TITLE-ABS-KEY (multimorbid*)
#10	INDEXTERMS (“multiple chronic conditions”)
#11	TITLE-ABS-KEY (“multiple chronic conditions”)
#12	INDEXTERMS (multimorbidity) OR TITLE-ABS-KEY (“multi morbid*”) OR TITLE-ABS-KEY (multimorbid*) OR INDEXTERMS (“multiple chronic conditions”) OR TITLE-ABS-KEY (“multiple chronic conditions”)
#13	INDEXTERMS (brazil)
#14	TITLE-ABS-KEY (brazil)
#15	TITLE-ABS-KEY (brazilian)
#16	INDEXTERMS (brazil) OR TITLE-ABS-KEY (brazil) OR TITLE-ABS-KEY (brazilian)
#17	(INDEXTERMS (prevalence) OR TITLE-ABS-KEY (prevalence) OR INDEXTERMS (epidemiology) OR TITLE-ABS-KEY (epidemiology) OR TITLE-ABS-KEY (surveillance)) AND (INDEXTERMS (multimorbidity) OR TITLE-ABS-KEY (“multi morbid*”) OR TITLE-ABS-KEY (multimorbid*) OR INDEXTERMS (“multiple chronic conditions”) OR TITLE-ABS-KEY (“multiple chronic conditions”)) AND (INDEXTERMS (brazil) OR TITLE-ABS-KEY (brazil) OR TITLE-ABS-KEY (brazilian))

**Table 4:** Web of Science search strategy.

Search	Query
#1	TS=(prevalence)
#2	TI=(prevalence)
#3	AB=(prevalence)
#4	TS=(epidemiology)
#5	TI=(epidemiology)
#6	AB=(epidemiology)
#7	TI=(surveillance)
#8	AB=(surveillance)
#9	#8 OR #7 OR #6 OR #5 OR #4 OR #3 OR #2 OR #1
#10	TS=(multimorbidity)
#11	TI=(multimorbid*)
#12	AB=(multimorbid*)
#13	TS=(“multiple chronic conditions”)
#14	#10 OR #11 OR #12 OR #13
#15	TS=(Brazil)
#16	TI=(brazilian)
#17	AB=(brazilian)
#18	#15 OR #16 OR #17
#19	#9 AND #14 AND #18

**Table 5:** Embase search strategy.

Search	Query
#1	prevalence/de AND [embase]/lim
#2	prevalence:ti,ab,kw AND [embase]/lim
#3	epidemiology/de AND [embase]/lim
#4	epidemiology:ti,ab,kw AND [embase]/lim
#5	surveillance:ti,ab,kw AND [embase]/lim
#6	#1 OR #2 OR #3 OR #4 OR #5
#7	multimorbidity/de AND [embase]/lim
#8	multimorbidity:ti,ab,kw AND [embase]/lim
#9	multimorbid*:ti,ab,kw AND [embase]/lim
#10	‘multiple chronic conditions’:ti,ab,kw AND [embase]/lim
#11	#7 OR #8 OR #9 OR #10
#12	brazil/de AND [embase]/lim
#13	brazil:ti,ab,kw AND [embase]/lim
#14	brazilian:ti,ab,kw AND [embase]/lim
#15	#12 OR #13 OR #14
#16	#6 AND #11 AND #15

**Table 6:** LILACS search strategy.

Search	Query
#1	(mh:(prevalence)) OR (prevalence) OR (mh:(epidemiology)) OR (epidemiology) OR (surveillance))
#2	(mh:(multimorbidity)) OR (multimorbid*) OR (“multi morbid*”) OR (mh:(“multiple chronic conditions”)) OR (“multiple chronic conditions”)
#3	(mh:(brazil)) OR (brazil) OR (brazilian)
#4	(mh:(prevalence OR epidemiology)) OR (prevalence OR epidemiology OR surveillance) AND (mh:(multimorbidity OR “multiple chronic conditions”)) OR (“multiple chronic conditions” OR multimorbid* OR “multi morbid*”) AND (mh:(brazil)) OR (brazil OR brazilian)

**Table 7:** SciELO search strategy.

Search	Query
#1	(ti:(prevalence)) OR (ab:(prevalence)) OR (ti:(epidemiology)) OR (ab:(epidemiology)) OR (ti:(surveillance)) OR (ab:(surveillance)) Filters applied: (Collections: Brazil)
#2	(ti:(multimorbid*)) OR (ab:(multimorbid*)) OR (ti:(“multiple chronic conditions”)) OR (ab:(“multiple chronic conditions”)) Filters applied: (Collections: Brazil)
#3	((ti:(prevalence)) OR (ab:(prevalence)) OR (ti:(epidemiology)) OR (ab:(epidemiology)) OR (ti:(surveillance)) OR (ab:(surveillance))) AND ((ti:(multimorbid*)) OR (ab:(multimorbid*)) OR (ti:(“multiple chronic conditions”)) OR (ab:(“multiple chronic conditions”))) Filters applied: (Collections: Brazil)

Besides the database search, additional studies might be retrieved by manually reviewing the reference lists of included studies and relevant reviews in the field.

### Selection process

**Fig 1:**
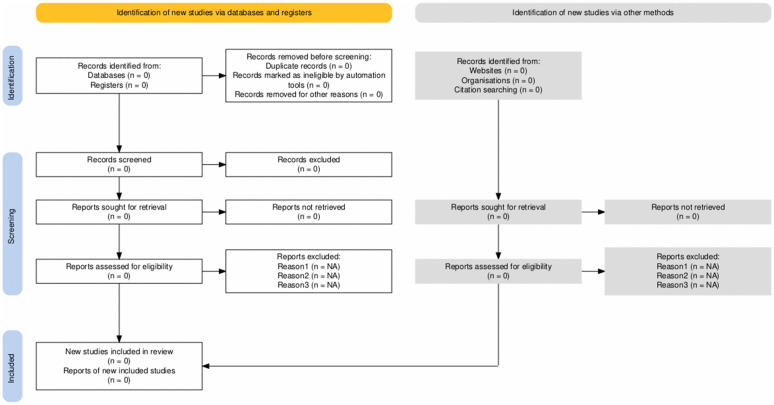
PRISMA 2020 Flow Diagram for Systematic Reviews doi:10.1371/journal.pone.0150625.g001.

A single reviewer (CCP) has conducted the initial search, identified, and removed duplicates using Rayyan [38]. Two reviewers (CCP and LSM) independently screened the titles and abstracts of studies retrieved during the searches to identify relevant articles. The two reviewers are now assessing the full texts of studies as potentially eligible according to the inclusion and exclusion criteria in parallel. Inter-reviewer agreement will be assessed using Cohen’s kappa coefficient (κ), with a minimum threshold of 0.75 indicating high agreement [39]. Discrepancies will be resolved by consensus or consultation with a third reviewer (RAG). The study selection process will be documented in a PRISMA flow diagram [40] (Fig 1).

### Data extraction

Two reviewers (CCP and LSM) will independently extract data using a standardized form in Research Electronic Data Capture (REDCap) [41]. A pilot test involving five manuscripts will be conducted to ensure consistency. The following data will be collected: author(s), year of publication, study design (cross-sectional, cohort baseline), study location (municipality, state, region, national), study year, number of chronic conditions assessed, list of chronic conditions included, type of condition measurement (self-reported, measured, or a combination of both), definition of MM if applicable (≥2, ≥ 3, ≥ 4, or ≥5 chronic conditions), demographic characteristics of participants (age group and sex), and number of participants (total and with MM). Any discrepancies in data extraction will be resolved by consensus or by a third reviewer (RAG). In cases of missing or unclear data, study authors will be contacted via email up to three times.

### Risk of bias assessment

Methodological quality or risk of bias will be assessed using the Joanna Briggs Institute (JBI) Checklist for prevalence studies. This tool evaluates nine criteria, with each item rated as “Yes,” “No,” “Unclear,” or “Not Applicable.” The following JBI items are used to measure methodological quality: 1. Was the sample frame appropriate to address the target population?; 2. Were study participants sampled in an appropriate way?; 3. Was the sample size adequate?; 4. Were the study subjects and the setting described in detail?; 5. Was the data analysis conducted with sufficient coverage of the identified sample?; 6. Were valid methods used for the identification of the condition?; 7. Was the condition measured in a standard, reliable way for all participants?; 8. Was there appropriate statistical analysis?, and 9. Was the response rate adequate, and if not, was the low response rate managed appropriately? The literature does not indicate a standardized cutoff point for classifying studies, with the following classification being most commonly adopted: ≥ 70% of JBI items met: study with low risk of bias; 50–69% of items met: study with moderate risk of bias and <50% of criteria met: study with high risk of bias [35].

The assessment will be conducted independently by two reviewers (CCP and LSM). Discrepancies between the reviewers will be resolved by consensus or consultation with a third reviewer (RAG).

### Evidence synthesis and meta-analysis

Study characteristics will be summarized descriptively in tables and narrative text. For meta-analysis, pooled prevalence estimates will be calculated using random-effects models with Restricted Maximum Likelihood (REML) estimators. Results will be presented as forest plots with 95% confidence intervals (CIs) [42]. Heterogeneity will be assessed using Cochran’s Q test (p-value<0.10) [43] and the I² statistic (≥50% indicating substantial heterogeneity) [43]. Subgroup analyses will explore potential sources of heterogeneity, including sex, age group, region of Brazil, number of chronic conditions included, sample size, study year, and evidence quality. Publication bias will be evaluated using funnel plots and Egger’s test [44]. All analyses will be conducted using the “meta” [45] and “metafor” [46] packages in R software version 4.2.2 [45].

## Discussion

This study will conduct a systematic review and meta-analysis to estimate the prevalence of MM among Brazilian adults aged 18 years and older in community settings. Synthesizing this evidence is crucial for informing public health policies and developing preventive strategies to reduce Brazil’s MM burden, particularly given the country’s concurrent epidemiological and nutritional transitions, accelerated population aging, and potential disparities in MM prevalence across age groups, sexes, and geographic regions.

While a previous Latin American meta-analysis included Brazilian data, it had three notable limitations that our study addresses: (1) it did not calculate Brazil-specific pooled prevalence estimates, (2) it lacked subgroup analyses by age, sex, or region, and (3) it excluded data published after 2017 [22]. Our comprehensive assessment will provide vital evidence to strengthen policies guiding MM-related health promotion, disease prevention, surveillance, and integrated care. Crucially, our demographic subgroup analyses will enable the development of targeted interventions tailored to specific population needs.

Although this study is limited to Brazil’s adult population, its potential findings may hold relevance for other low- and middle-income countries undergoing similar epidemiological, nutritional, and demographic transitions, particularly in Latin America. The subgroup analysis – especially by sex and age group – will help identify populations most vulnerable to MM and inform public health policies. Specifically, the results may serve as a reference for developing: (1) health promotion strategies, (2) disease prevention approaches, (3) health surveillance systems, and (4) comprehensive care models for individuals with MM across different settings. Furthermore, this study may stimulate further research to assess the MM burden in diverse countries, thereby advancing global knowledge on this critical health challenge.

Due to its significant impact on population health and healthcare systems worldwide, MM has gained increasing recognition [10]. Therefore, by using a comprehensive search approach and conducting subgroup analyses, this systematic review and meta-analysis aims to generate comparable estimates. To the best of our knowledge, this is the first protocol designed to conduct a meta-analysis and systematic review with this objective in Brazil. Once the results are published, the study’s limitations and implications will be thoroughly discussed.

## Supporting information

S1 File PRISMA-P 2015 Checklist. (DOCX)
